# Oxidative Stress and Dietary Antioxidants in Head and Neck Cancer

**DOI:** 10.3390/antiox14050508

**Published:** 2025-04-24

**Authors:** A Jeong You, Jaehyung Park, Jae-Min Shin, Tae Hoon Kim

**Affiliations:** 1 Department of Otorhinolaryngology-Head and Neck Surgery, College of Medicine, Korea University, Seoul 02841, Republic of Korea; ajungyi@naver.com (A.J.Y.); soonsliy131@gmail.com (J.P.); shinjm0601@korea.ac.kr (J.-M.S.); 2 Mucosal Immunology Institute, College of Medicine, Korea University, Seoul 02841, Republic of Korea

**Keywords:** oxidative stress, reactive oxygen species (ROS), antioxidants, dietary antioxidants, head and neck squamous cell carcinoma (HNSCC)

## Abstract

Oxidative stress serves as both a driver and result of redox metabolism across diverse physiological and pathological states, including cancer. Head and neck squamous cell carcinoma (HNSCC), the sixth most prevalent malignancy worldwide, is no exception. HNSCC is strongly linked to modifiable external risk factors such as tobacco smoking, alcohol consumption, and high-risk human papilloma (HR-HPV) infection. These risk factors are associated with elevated oxidative stress, which contributes to carcinogenesis through DNA damage, chronic inflammation, and dysregulation of cell signaling pathways. Current treatment options for HNSCC have limitations and burden of side effects. Studies have been conducted on potent dietary antioxidants for the prevention and adjunctive treatment of HNSCC. This review aims to explore the contribution of oxidative stress to carcinogenesis in general and the three major risk factors for HNSCC. We evaluate latest evidence for nine dietary antioxidants such as vitamin C, vitamin E, carotenoids, epigallocatechin-3-gallate (EGCG), and curcumin, that have shown promise in preclinical and clinical studies. We discuss how these compounds mitigate ROS, influence cancer-related signaling pathways, and modulate tumor microenvironment. Despite encouraging findings, current clinical data remain limited and inconclusive, highlighting the need for further research on possible dietary antioxidants for HNSCC.

## 1. Introduction

Head and neck cancer (HNC) is a heterogeneous group of malignancies arising from the mucosal epithelium of the oral cavity, pharynx, and larynx. Among HNCs, head and neck squamous cell carcinoma (HNSCC) accounts for more than 90% of cases and represents a significant global health burden. HNSCC is the sixth most common cancer worldwide, with approximately 870,000 new cases and 440,000 deaths annually. The disease primarily affects individuals over the age of 50 and is more prevalent in men, with geographic variation influenced by cultural and environmental exposures. Compared to many other cancers, HNSCC has relatively well-established risk factors. Alcohol consumption, tobacco smoking, and high-risk human papilloma virus (HR-HPV) infection—particularly HPV type 16—are recognized as the primary etiological factors [1]. These factors not only increase the likelihood of developing HNSCC but also influence the molecular and clinical characteristics. While tobacco- and alcohol-associated HNSCCs are often linked to extensive genetic alterations and poorer outcomes, HPV-related HNSCC tends to occur in younger patients and is generally associated with a more favorable response to therapy [2]. Interestingly, these risk factors converge on a shared biological mechanism: the induction of oxidative stress. Oxidative stress results from a disrupted redox equilibrium between reactive oxygen/nitrogen species (ROS/RNS) and the antioxidant protection mechanism against them [3]. This imbalance, whether caused by increased ROS/RNS production or diminished antioxidant capacity, contributes to a wide range of pathological states [4], including the development and progression of HNSCC. Excessive ROS levels can promote DNA damage, chronic inflammation, and aberrant cell signaling—processes that are key drivers of carcinogenesis. Given this metastatic link, there is growing interest in the role of dietary antioxidants as potential modulators of oxidative stress and supportive agents in cancer prevention and therapy. As advanced HNSCC often carries a poor prognosis and current treatment options remain limited, further investigation into preventive and adjunctive strategies is needed—particularly in relation to oxidative stress.

Although existing clinical data on the role of dietary antioxidants in HNSCC remain limited and largely inconclusive, the biological plausibility and public health relevance of this topic warrant a critical and integrative review. By acknowledging both the promise and the current limitations of available evidence, this review seeks to provide a balanced perspective that may inform future research and clinical considerations.

In this review, we aim to investigate how oxidative stress contributes to the initiation and progression of HNSCC and to evaluate the current evidence on the potential dietary antioxidants in its prevention and treatment. To date, few reviews have comprehensively examined the intersection of oxidative stress and HNSCC through the lens of its primary external risk factors—tobacco, alcohol, and HR-HPV infection. This review offers a mechanistic perspective on how these etiological agents may promote redox imbalance and drive carcinogenesis. Moreover, unlike previous reviews that narrowly focus on individual antioxidants, our approach provides a broader yet focused synthesis, specifically tailored to HNSCC subsites classified by ICD codes. We critically evaluate nine dietary antioxidants based on both preclinical and clinical evidence, offering both mechanistic insights as well as practical relevance for future research and clinical strategies in HNSCC management.

## 2. Oxidative Stress in Cancer

### 2.1. Overview of Oxidative Stress

Oxidative stress, a term first defined by Helmut Sies in 1985, refers to “an imbalance between oxidants and antioxidants in favor of oxidants, leading to the disruption of redox signaling and control and/or molecular damage” [3]. Oxidants are an inevitable byproduct of oxidation–reduction (redox) reactions occurring in living cells, and aerobic metabolism for energy production generates diverse ROSs. Some ROSs are free radicals, which are atoms or molecules possessing one or more unpaired electrons within an atomic or molecular orbital. Other ROSs are nonradicals that have all their electrons paired [5]. The two primary endogenous sources of ROSs are mitochondria and nicotinamide adenine dinucleotide phosphate oxidases (NOXs). Mitochondria generate superoxide as a byproduct of aerobic metabolism, and NOXs generate superoxide in response to various intrinsic and extrinsic stimuli [6]. ROSs are highly reactive molecules capable of damaging nucleic acids, lipids, and proteins, leading to alterations in their functions [7]. Therefore, cells possess a defense system called the antioxidant network, which maintains ROSs at physiological levels [8]. Antioxidants can be endogenously produced within the body, acquired through diet, or artificially synthesized. Endogenous antioxidants include enzymatic antioxidants, such as superoxide dismutase (SOD), glutathione peroxidase (GPx), catalase, and peroxiredoxin (Prx). Nonenzymatic antioxidants such as reduced glutathione (GSH) and coenzyme Q10 (CoQ) are endogenous antioxidants. Dietary antioxidants include vitamins C and E, phenolic compounds, ergothionein, and carotenoids. Clinically used synthetic antioxidants include ebselen (2-phenyl-1,2-benzoisoselenazol-3(2H)-one), edaravone, and N-acetylcysteine [5]. The damage caused by ROS has been established as a contributing factor to various chronic diseases such as neurodegenerative diseases, emphysema, cardiovascular and inflammatory diseases, cataracts, and malignancy [4].

### 2.2. Role of Oxidative Stress in Cancer

Oxidative stress plays a critical role in driving and sustaining key cancer hallmarks, such as deoxyribonucleic acid (DNA) damage, angiogenesis, cell cycle progression, and metastatic ability. ROSs interact with DNA molecules, causing damage such as single-strand breaks, double-strand breaks, and base modifications. ROSs, such as nitric oxide, interfere with the repair mechanisms of DNA, such as direct reversal and base excision repair [9]. Uncontrolled DNA damage leads to genomic instability in healthy cells and cancer [10]. During the early stages of tumor initiation, autophagy removes damaged organelles and cells, thereby decreasing ROS levels and exerting tumor-suppressive effect [11,12]. Excessive ROS levels interfere with autophagy [13]. At this stage, deletion of autophagy-related genes (*ATG*) 5 and 7 results in autophagy inhibition, leading to the accumulation of oxidative stress, damaged tissues, and inflammation, all of which favor tumor initiation [12,14]. Mutual interactions between chronic inflammation and cancer have been clearly identified. Chronic inflammation results in a significant number of immunosuppressive cells and release of cytokines, leading to ROS generation This is associated with signaling pathways like nuclear factor kappa-light-chain-enhancer of activated B cells (NF-κB), and overexpression of ROS in turn worsens chronic inflammation. Excessive ROS activate the mitogen-activated protein kinase (MAPK) signaling pathway, which phosphorylates and activates activator protein-1 (AP-1) components such as c-Jun and c-Fos. AP-1 and oxidative stress have a mutual relationship, and dysregulated AP-1 expression contributes to various diseases, including lung cancer [15]. Researchers have found that NF-κB may contribute to tumor cell survival by increasing the expression of antiapoptotic genes, such as B-cell lymphoma2 (*BCL2*), which suppresses apoptosis. Moreover, NF-κB enhance the expression of hypoxia-inducible factor-1α (HIF-1α), contributing to cellular resistance against stress and hypoxic conditions [16]. Apoptosis dysregulation is a defining characteristic of cancer. Apoptosis in carcinogenesis is inhibited through the activation of NF-κB, which upregulates the production of antiapoptotic proteins such as BCL-XL (B-cell lymphoma XL), BFL1 (a BCL-2-related protein), and GADD45β (growth arrest and DNA-damage-inducible 45β). These proteins promote cell survival by counteracting pro-apoptotic signals. Increased ROS levels inactivate phosphatase and tension homolog (PTEN) and upregulate the phosphoinositide 3-kinase/protein kinase B (PI3K/Akt) signaling pathway. The Akt pathway inactivates proapoptotic transcription factors BCL2-associated agonist of cell death (BAD) and BCL2-associated X protein (BAX), resulting in cell proliferation. Elevated ROS generation is also associated with the protein kinase D (PKD) signaling pathway. Specifically, PKD1 enhances cell viability by downregulating the pro-apoptotic c-Jun N terminal protein kinase (JNK) pathway and upregulating the pro-survival transcription factor NF-κB. This dual regulatory action promotes the resistance of cancer cells to oxidative stress and apoptosis [13].

As described above, ROS may play a role in preventing damaged cells from undergoing apoptosis after DNA damage, thereby promoting their transformation into cancer cells. After developing into cancer cells, ROS drive cancer cell proliferation through multiple pathways. As mentioned earlier, the PI3K/Akt signaling pathway is activated by elevated ROS levels. PI3K/Akt activate downstream mammalian target of rapamycin (mTOR) and HIF-1α, promoting angiogenesis, which is important for cancer cell survival in a hypoxic environment [17]. In the advanced phases of cancer progression, cancer cells induce oxidative stress, activating transcription factors HIF-1α and NF-κB. This promotes autophagy of stromal cells in the tumor microenvironment, supporting tumor growth by supplying nutrients-rich metabolites such as lactate and ketones [12]. Increased ROS levels induce the inhibition of the cell division cycle 14B (CDC14B) phosphatase, leading to the upregulation of cyclin-dependent kinase1 (CDK1). CDK1 contribute to the transition from G2 to M phase of the cell cycle. Unchecked activity can drive the cell cycle forward even in the presence of DNA damage, thereby contributing to cancer cell progression [13,18]. ROS also promote metastasis and the epithelial-to-mesenchymal transition (EMT) process. EMT contributes to cancer evolution by enhancing tumor invasion, proliferation, and metastasis. During the EMT, epithelial cells undergo a loss of adhesion properties and gain mesenchymal traits, thereby increasing their motility and invasiveness [19]. ROSs, such as H_2_O_2_, elevate the expression or activity of proteins, such as matrix metalloproteinases (MMPs), vascular endothelial growth factor (VEGF), epidermal growth factor (EGF), and EGF receptor (EGFR), which are crucial in tumor metastasis [20]. In summary, oxidative stress is thought to be involved in multiple stages of cancer development, from the accumulation of DNA damage in healthy cells that may lead to malignant transformation to the subsequent proliferation and metastasis of cancer cells.

## 3. Oxidative Stress and Dietary Antioxidants in HNSCC

### 3.1. Overview of HNSCC

HNC ranks as the sixth most common malignancy globally, with more than 870,000 new diagnoses and 440,000 associated deaths reported in 2020. HNSCC constitutes approximately 90% of HNC cases [1]. HNSCC presents significant treatment challenges and requires a multidisciplinary approach. Surgery, radiotherapy, and systemic therapy constitute the cornerstone of treatment for locally advanced disease. The advent of immunotherapy in the conventional treatment of recurrent or metastatic HNSCCs has significantly revolutionized their management. However, much is yet to be understood, and HNSCC remains a highly complex disease. Patients with stage III or higher HNSCC generally have a poor prognosis. For instance, the 5-year survival rate of locoregionally advanced laryngeal cancer is approximately 40% [21], and over 60% of HNSCC patients are diagnosed at stage III or IV disease [22]. Moreover, HNSCC imposes a substantial socioeconomic burden due to the high costs associated with its diagnosis, treatment, and long-term management, as well as the loss of productivity due to high morbidity and mortality [23].

The major risk factors for HNSCC are tobacco smoking, alcohol intake, and high-risk human papillomavirus (HR-HPV) infection [1]. HNSCC, which is traditionally linked to heavy tobacco and alcohol use, is typically diagnosed in older individuals and is gradually declining worldwide owing to reduced tobacco consumption. In contrast, the incidence of HPV-associated oropharyngeal cancer, mainly caused by HPV type 16, is increasing, particularly among young populations in North America and northern Europe [22]. These risk factors associated with HNSCC are difficult to eliminate. A pooled analysis of 30 case–control studies related to drinking or smoking cessation revealed that it may take up to 20 years or more for the risk of HNSCC to reach that of never drinkers or smokers [24]. Moreover, HPV infections are irreversible. Therefore, more chemopreventive measures are needed rather than simply trying to avoid these risk factors. Changes in redox metabolism are thought to be involved in all stages of HNSCC through cancer etiology, progression, therapy, and quality of life after treatment [25]. Therefore, we reviewed the involvement of oxidative stress in the three major risk factors of HNSCC and the possible dietary antioxidants that patients can easily access. For this review, we classified HNSCCs into cancer of oral cavity, oropharynx, hypopharynx, oral cavity or pharynx not otherwise specified, or larynx or HNSCC unspecified. Cancers of the salivary glands, nasal cavity, ear, paranasal sinuses, and esophagus were not included owing to differences in epidemiology and pathology. The subsites were classified based on disease codes from the International Classification of Diseases (ICD)-10 [26].

### 3.2. Oxidative Stress in HNSCC

The three primary risk factors for HNSCC are tobacco smoking, alcohol consumption, and HR-HPV infection [1]. When these risk factors are combined, there are devastating results. For instance, a pooled analysis of 17 case–control studies (11,221 cases and 16,168 controls) demonstrated that the combined effect of alcohol and tobacco use was greater than a multiplicative effect on HNSCC risk [27]. HNSCC has relatively distinct external risk factors compared with other cancers. These risk factors contribute to cancer development through various mechanisms; however, they share the common feature of being associated with oxidative stress, which is crucial in carcinogenesis. Therefore, we focused on how alcohol consumption, tobacco smoking, and HR-HPV infection contribute to the development of HNSCCs related to oxidative stress.

Ethanol is metabolized to the carcinogen acetaldehyde by NADPH-dependent cytochrome P450 2E1 (CYP2E1), alcohol dehydrogenase (ADH), and catalase. Induction of CYP2E1 affects the cellular oxidative balance by generating ROS, leading to the oxidation of proteins, lipids, and DNAs [28]. Tobacco smoking is attributed to 42% of HNSCC-related deaths [25]. Tobacco smoke can be classified into two phases, namely gas and tar (particulates). The gas phase occurs during the combustion of tobacco, and consists of chemicals smaller than 0.1 μm. The tar phase contains compounds with diameters ranging from 0.1–1 μm, averaging 0.2 μm. Both phases produce a significant number of free radicals [29]. A recent preclinical study on HPV-negative HNSCC cell lines revealed the molecular mechanism by which ROS produced by alcohol and tobacco smoking contribute to the development of HNSCC. Oxidative stress induced by tobacco and alcohol induces the dimerization of transmembrane 4 L6 family member 19 (TM4SF19) in the endoplasmic reticulum. This dimerization prevents guanine and adenine-binding protein β1 (GABPβ1) from proteasomal degradation, and GABP transcription factor complex promotes Yes-associated protein 1 (YAP1) transcription. YAP, a key effector of the Hippo-YAP signaling pathway, is frequently dysregulated in various human cancers. Increased YAP activity is strongly associated with dismal prognosis and therapeutic resistance in multiple cancers, including HNSCC [30].

HPV infection and oxidative stress have been suggested to influence each other in a bidirectional manner, potentially acting as both causes and consequences depending on the context. Studies have revealed that oxidative stress affects early stages of viral infection by altering the local redox environment and facilitating viral integration by enhancing DNA damage and weakening repair mechanisms via HPV oncoproteins such as E6 and E7. Moreover, high levels of oxidative stress markers, such as ferritin, are associated with reduced clearance of HPV infection [31]. HPV induces ROS production via multiple pathways. HPV infection drives chronic inflammation through various mechanisms, leading to oxidative stress. Compared to HPV-negative HNSCC, HPV-positive HNSCC demonstrates increased levels of β-oxidation associated genes. β-oxidation is a fatty acid oxidation in mitochondria, which can generate ATP energy more efficiently than glycolysis (108 ATP molecules from oxidation of palmitoyl-CoA compared with 32 ATP molecules from oxidation of glucose). β-oxidation produces H_2_O_2_, which consequently induces more ROS, such as superoxide radical (O_2_^•−^). O_2_^•−^ diffuses into the cytosol and produces hydroxyl radicals (•OH) by Fenton and Haber-Weiss reactions. In turn, •OH trigger oxidative damage by inducing malondialdehye (MDA) and 4-hydroxynonenal (4-HNE) [32]. MDA is a marker of oxidative stress and its elevation is associated with tumor extent and unfavorable prognosis [33].

In addition, the viral proteins E6 and E7 induce nicotinamide adenine dinucleotide phosphate oxidase 2 (NOX2)-dependent ROS generation, leading to oxidative DNA damage. ROS-mediated genomic instability is more apparent in HPV-positive HNSCC than in HPV-negative HNSCC [6]. Oncogenic protein E6 also inactivates antioxidants such as SOD2 and GPx, leading to increased oxidative DNA damage [34]. E6 and E7 oncoproteins promote p53 and retinoblastoma protein (pRb) degradation, which is thought to be one of the key mechanisms by which HPV oncoproteins induce genomic instability [35]. In summary, HR-HPV infection leads to ROS generation by multiple pathways such as inducing chronic inflammation, β-oxidation, NOX2 activation, and decreasing antioxidants. ROSs, in turn, cause oxidative DNA damage, which can be detected by measuring the level of 8-oxo-7,8-dihydro-2′-deoxyguanosine (8-oxo-dG). Guanine has the lowest redox potential among the four DNA bases, making it the most prone to oxidation. Its oxidized form, 8-oxo-dG, has an even lower redox potential, rendering it highly susceptible to further oxidation. Therefore, 8-oxo-dG is commonly recognized as a biomarker of oxidative stress [9,29]. In summary, the three major risk factors for HNSCC are closely related to oxidative stress, highlighting the importance of maintaining the redox balance for the prevention and treatment of HNSCC.

However, numerous studies have revealed that components of the antioxidant defense system, such as paraoxonase-2 (PON2) and the Nuclear Factor Erythroid 2-Related Factor 2/Kelch Like ECH Associated Protein 1 (NRF2/KEAP1) signaling axis, can paradoxically support tumor progression and therapy resistance in HNSCC. PON2, a membrane-bound enzyme with antioxidant properties, is overexpressed in HNSCC tissues [36] and has been shown to promote tumor cell survival by mitigating mitochondrial ROS production and preventing apoptosis [37]. High PON2 expression was associated with decreased overall survival (Hazard Ratio = 1.53, 95% CI: 1.16–2.23, *p* = 0.0025) based on The Cancer Genome Atlas data [36], and has also been linked to resistance to both radiotherapy and chemotherapy [38]. Similarly, NRF2, a regulator of cellular redox homeostasis, is known to be constitutively activated in several cancers, including HNSCC. Under normal physiological conditions, NRF2 is tightly regulated by its cytoplasmic inhibitor KEAP1. However, dysregulation of this pathway can lead to persistent NRF2 activation, which facilitates tumor cell proliferation, metabolic rewiring, and evasion of oxidative-stress-induced apoptosis [39]. Furthermore, NRF2/KEAP1 signaling has been linked to cisplatin and radiotherapy resistance in HNSCC models [40,41]. These findings highlight that while antioxidant pathways play certain roles in protecting normal tissues, their dysregulation in cancer cells may confer selective advantages, contributing to malignant behavior and treatment resistance. Given these complex and sometimes opposing roles, the impact of oxidative stress on HNSCC remains debated and context-dependent. Some antioxidant mechanisms may suppress carcinogenesis under certain conditions, whereas others—particularly when overactivated—may paradoxically promote tumor survival and progression. Despite these discrepancies, a growing number of studies suggest that specific dietary antioxidants may provide chemopreventive or therapeutic benefit in subsets of HNSCC patients. In light of this, our review aims to provide an overview of nine dietary antioxidants that have shown mechanistic or clinical relevance, while also acknowledging the limitations and duality of antioxidant biology in cancer.

### 3.3. Dietary Antioxidants in HNSCC

As described above, impaired redox balance contributes to the development of HNSCC, from etiology to treatment [25]. In addition, it is very costly to treat HNSCC, and there are clear limitations to current therapies. Hence, prevention and increasing the efficiency of treatment are important. Dietary antioxidants that may have chemopreventive or adjuvant therapeutic roles in HNSCC have been investigated. Currently, no antioxidants are routinely used as therapeutic agents for HNSCC. In addition, research to date has not proven that any antioxidants are effective in preventing second primary malignancies in patients with HNSCC [42]. However, there have been many studies on the prevention of primary cancer and the alleviation of the side effects of chemotherapy and radiation therapy. One of the most referenced large-scale prospective cohort studies on diet and cancer is the NIH-AARP (National Institutes of Health–American Association of Retired Persons) Diet and Health Study. This study collected dietary and health data from over 500,000 participants through annual questionnaires, cancer registries, and national death indexes. It has provided valuable epidemiological insights, including analyses on dietary pattern in relation to HNC incidence. However, the NIH-AARP study relies heavily on self-reported dietary data, which introduces recall bias and limits the ability to establish causal relationships. Additionally, due to its study design, it cannot assess the effects of specific antioxidant compounds but instead categorizes dietary patterns broadly, such as “fruit and vegetable” [43] or “dietary fiber and grain” [44] intake. Therefore, while its findings are hypothesis-generating, they are not conclusive. Despite the limitations of large cohort studies, their findings support further investigation of individual antioxidant compounds, which we explore in detail in the following sections. We consider nine dietary antioxidants that have promising cancer preventive and/or therapeutic potential (Table 1).

Vitamin C is a potent aqueous-soluble electron donor in humans. Two pooled analysis studies by the INHANCE (International Head and Neck Cancer Epidemiology) Consortium, of supplement intake [45] and natural food intake [46], suggested an negative association between vitamin C intake and HNSCC incidence. A recent case–control study of 101 patients also reported corresponding results [47]. A large prospective cohort study in the Netherlands confirmed these results, especially for oral-cavity cancer, compared with oro/hypopharyngeal and laryngeal cancers. As daily vitamin C intake increased, the relative risk of HNSCC gradually declined. The highest vitamin C dose in the study was 144.8–153.3 mg/day, which was associated with 61% reduced risk for HNSCC, compared to 55.2–63.5 mg intake per day [48]. However, none of these studies offered a specific tolerable dose of vitamin C, and no statistically clear dose–response relationship was observed [45]. Moreover, most studies rely on observational or case–control designs with self-reported dietary data, introducing potential bias and limiting causal inference. For instance, one study reported that high citrus fruit intake was linked to an elevated risk of HPV16-associated HNSCC. In addition, the risk was greatly reduced in subjects with low citrus fruit exposure and polymorphisms in the *SLC23A2* allele, which encodes the sodium-dependent vitamin C transporter (SVCT2). This report suggested that high dietary consumption of vitamin C may exhibit paradoxical impacts on HNSCC in certain situations and genetic backgrounds [49]. Moreover, a meta-analysis conducted by Patini et al., following PRISMA guidelines, found no statistically significant association between vitamin C intake and the risk of oral cavity carcinoma [50]. Therefore, although some studies suggest that vitamin C may play a preventive effect against HNSCC, further research is needed to determine its optimal use, accounting for individual variability and study design limitations.

Vitamin E is a fat-soluble antioxidant including four tocopherols and four tocotrienols: α-, β-, γ-, and δ-tocopherol and α-, β-, γ-, and δ-tocotrienol. In a pooled analysis of 10 case–control studies in the INHANCE consortium, vitamin E intake showed an inverse association with HNSCC subtypes [51]. However, in a double-blind, placebo-controlled randomized controlled trial (RCT) involving 540 patients with HNSCC, patients receiving long-term supplementation with 400 IU of vitamin E per day had higher all-cause mortality [52] and a higher rate of recurrence or second primary malignancy [53]. These results may be due to the paradoxical pro-oxidant effect at high doses [54]; therefore, long-term use of high doses requires caution. Vitamin E also has positive effects on patients undergoing radiation therapy. α-Tocopherol failed to alleviate severe radiation-induced side effects when administered as a supplement [55]. However, when combined with pentoxifylline, which blocks the production of the inflammatory marker TGF-β1, vitamin E reduced oral mucositis (OM) and dysphagia [56]. Moreover, when combined with vitamin C, it also reduced radiotherapy-induced xerostomia [57] Besides oral administration, when patients were assigned to rinse the oral cavity with a vitamin E-containing oil solution, it reduced radiation-induced OM [58]. Hence, vitamin E can be an effective measure for attenuating the side effects induced by radiotherapy, with appropriate usage and in combination with other nutrients. α-Tocopherol is also known to reduce the toxicity of vitamin A analogs. Vitamin A analogs have been actively studied over the last few decades. However, their use is limited owing to the short duration of response, resistance, and toxicities such as cheilitis, dry skin, and conjunctivitis [59,60]. One vitamin A analog is 13-cis-retinoic acid (13-cRA), which has shown promising results in HNSCC treatment when combined with α-tocopherol and interferon-α (IFN-α). In a phase-II bioadjuvant trial of patients with locally advanced HNSCC, 86% of patients successfully completed the 12-month treatment with a 13-cRA, IFN-α, and α-tocopherol combination, and it was generally well tolerated with promising results [61]. In the long-term follow-up, the combination demonstrated significant efficacy in preventing both recurrence and development of second primary tumors. The 5-year overall survival was 81.3% (95% confidence interval (CI), 63.7–90%), which was significantly higher than the historical 5-year overall rate for advanced HNSCC (approximately 40%) [62].

Carotenoids (CTDs) are a broad group of tetraterpenoids that are abundant in yellow to red fruits and vegetables. In human plasma, α-carotene, β-carotene, lycopene, lutein, zeaxanthin, and β-cryptoxanthin constitute more than 95% of the total CTDs. Nutrients like α-, β-, and γ-carotene and β-zeacarotene is easily converted into vitamin A, while nonprovitamin A CTDs include lycopene, lutein, and zeaxanthine. CTDs effectively prevent ROS formation because of the long polyene chain of 8–13 conjugated double-bond structures [63]. In addition to their antioxidant properties, many in vitro studies have revealed chemopreventive properties of CTDs through their action in key intracellular tumor signaling processes, such as the ERK-MAPK and PI3K/AKT/mTOR pathways [64]. An important study with a considerable sample size on the relationship between CTDs and HNSCC is a pooled analysis of 10 case–control studies of 18,207 subjects in the INHANCE consortium by Leoncini et al. The authors showed a 39% risk reduction of oropharyngeal and laryngeal cancer in the highest quintile of CTDs intake compared to the lowest quintile of CTDs intake [65]. A meta-analysis of 15 case–control studies and one prospective cohort study found that intake of β-carotene equivalents was associated with a risk reduction of 46% (95% CI 20–63%) for oral cancer, and 57% (95% CI 23–76%) for cancer of the larynx. Lycopene and β-cryptoxanthin also reduced the risk of laryngeal cancer. Specifically, subjects in the highest category of carotenoid consumption experienced 50% (95% CI 11–72%) risk reduction for laryngeal cancer with lycopene and 59% (95% CI 49–67%) reduction with β-cryptoxanthin. Additionally, lycopene, α-carotene, and β-cryptoxanthin were linked to at least a 26% reduction (95% CI 2–4%) in the risk of oropharyngeal cancers, emphasizing their protective role in HNSCC prevention [66]. Edefonti et al. conducted a pooled analysis of five case–control studies of 2452 cases, and identified three dietary patterns. The pattern referred to as ‘antioxidant vitamins and fiber’ showed the highest levels for vitamin C, total carotene, and lutein. This dietary pattern showed an inverse association with oropharyngeal cancer (odds ratio (OR) = 0.57, 95% CI 0.43–0.76) when comparing the highest to the lowest score quintile [67]. De Vito et al. conducted a multi-study factor analysis of five studies analyzed by Edefonti et al. The authors reported corresponding results with a high intake of antioxidant vitamins, with the greatest loading on vitamin C and total carotene [68]. Although not specifically targeting CTDs, numerous studies with large sample sizes have also proven that large consumption of fruits and vegetables, which are rich in CTDs, is associated with lower risk for HNSCC [69,70,71]. Beyond cancer prevention, carotenoids have also been found to improve treatment outcomes in patients with HNSCC. An RCT of 540 patients demonstrated that carotenoids reduced the risk of local recurrence and alleviated the severe adverse effects of radiation therapy, indicating their dual roles in prevention and therapeutic support [55]. However, results across studies are inconsistent. For instance, a large cohort study in the Netherlands showed no notable association between intake of α-carotene, β-carotene, lutein plus zeaxanthin, or lycophene with overall risk of HNSCC [48]. This may be accounted for by many factors, such as heterogeneity in dietary sources, variations in study design, varying degrees of exposure to smoking and alcohol use, and differences in HNSCC subtypes.

Green tea is effective in various cancer types. Its efficacy is primarily associated with its polyphenol content, which includes epicatechin, epigallocatechin, epicatechin-3-gallate, and epigallocatechin-3-gallate (EGCG). Of these, EGCG is the predominant and has been studied for its chemopreventive role [72]. Polyphenols are also potent antioxidants and radical scavengers. The role of green tea in HNSCC prevention was supported by a case–control study of 147 patients in Iran, which used a standardized questionnaire. Compared to individuals who never consumed green tea, the risk of developing oral cancer for those using green tea was significantly reduced. Individuals who consumed less than a cup of green tea daily had an OR of 0.29 (0.16–0.52), while those who consumed one or more cups of green tea daily had an OR of 0.38 (0.17–0.86) [73]. Another case–control study involving 396 patients with HNSCC in Taiwan reported similar results. There was a 6% reduction in HNSCC risk in subjects who had a cup of green tea daily (OR = 0.94, 95% Cl, 0.90–0.98), while oolong and black tea consumption had no statistical association with HNSCC risk [74]. One mechanism underlying the anticancer activity of EGCG is the targeting of tyrosine kinase receptors, such as EGFR and VEGFR [75]. In a mouse xenograft model of HNSCC, EGCG augmented the cell-growth inhibition by the EGFR-tyrosine kinase inhibitor, erlotinib [76]. A phase Ib study of green tea polyphenon E and erlotinib in advanced premalignant lesions (APLs) of the oral cavity and larynx showed similar results. This combination resulted in pathologic responses in 17/19 patients, with 66.3% cancer-free survival and 93% overall survival [77]. When not administered with erlotinib, green tea extract showed positive results in patients with high-risk oral premalignant lesions. High doses of green tea extract resulted in clinical responses in 58.8% (750 and 1000 mg/m^2^) and 36.4% (500 mg/m^2^) of patients, whereas the placebo group had a clinical response of 18.2% [78]. In patients diagnosed with oral cancer, mouth rinsing with green tea improved oral health status [79]. In a xenograft model of HNSCC stem cells, EGCG inhibited the expression of stem cell markers by inhibiting the Notch pathway and augmented cisplatin-mediated chemosensitivity, demonstrating that EGCG is a promising dietary antioxidant with both chemopreventive and therapeutic potential [80]. However, these studies are based on small sample sizes or preclinical models; thus, their translation into clinical outcomes requires further validation in larger human trials.

Curcumin is a natural compound found in the rhizomes of *Curcuma longa* L. (turmeric) plants. Numerous preclinical studies have revealed the therapeutic potential of curcumin in the treatment of HNSCC. Curcumin exerts broad anti-HNSCC effects by targeting multiple cancer-driving pathways such as NF-κB, JAK/STAT, and EGFR. Curcumin modulates the tumor microenvironment not only by promoting cell cycle arrest, apoptosis, and cytotoxicity, but also targeting cancer-associated fibroblasts, immune responses, and lymphovascular niches [81]. These mechanisms contribute to its observed antiproliferative and antimetastatic activity and support its development as an adjunct to conventional therapies in HNSCC [82,83]. Despite its safety being recognized by the Food and Drug Administration (FDA), the clinical use of curcumin is hindered by its low bioavailability. Researchers have attempted to overcome this problem by developing curcumin analogs and novel drug delivery systems [81]. A clinical trial involving 15 patients demonstrated enhanced bioavailability of curcumin following the transmucosal administration of microgranular curcumin. Higher serum levels of curcumin decreased the serum levels of fibroblast growth factor-2, granulocyte macrophage colony-stimulating factor, and interleukin (IL)-17 in patients with HNSCC [84]. APG-157 is a botanical drug composed of multiple polyphenols, including curcumin. Its oral delivery showed systemic absorption, and decreased Bacteroidetes species and inflammatory markers such as IL-1β, IL-6, and IL-8 in the saliva of subjects with oral cancer. Immune T cells also increased in tumor tissue, highlighting the therapeutic potential of curcumin by improving its bioavailability [85]. Kim et al. also conducted a pilot study of 39 subjects (21 HNSCC patients, 13 patients with dental caries, and 5 healthy controls) and demonstrated that curcumin treatment significantly reduced the activity of IκB kinase β (IKKβ), which is known to promote cancer progression through activation of NF-κB pathway [86]. There has been little clinical research on curcumin in patients with HNSCC, but existing research has shown promising results. Kuriakose et al. conducted a phase IIb double-blind, placebo-controlled RCT with 223 patients with oral leukoplakia. Patients either had 3.6 g curcumin/day or a placebo for 6 months. The clinical response was better in the curcumin group (67.5%, 95% CI 58.4–75.6%) than that in the control group (55.3%, 95% CI 46.1–64.2%). The combined clinical and histologic response was also better in curcumin group (hazard ratio 0.50, 95% Cl 0.27–0.92), without any safety concern [87]. Zhang et al. conducted a meta-analysis of six RCTs on the preventive and therapeutic effects of curcumin on treatment-related OM in patients with HNSCC. Curcumin did not lower the incidence of OM but lowered the incidence of severe OM and decreased its mean severity. It also considerably reduced weight loss [88].

Quercetin is a plant flavonol belonging to the flavonoid polyphenol group. Quercetin has been actively studied, especially regarding its antitumor effect in targeting the apoptotic pathway in oral squamous cell carinoma (OSCC) cell lines. A recent in vitro study has shown that quercetin induces ferroptosis by inactivating the mTOR/S6KP70 cascade and inhibiting cell growth in OSCC [89]. Quercetin induces G1 cell cycle arrest and apoptosis in OSCC cells by activating the p38 MAPK signaling pathway regardless of *TP53* mutation status [90]. Moreover, apoptosis is also induced by JNK-activation-regulated ERK1/2 and GSK3-α/β-mediated mitochondria-dependent apoptosis signaling pathway in tongue squamous cell carcinoma [91]. In addition to the apoptotic pathway, quercetin targets other pathways associated with cancer cell proliferation. In studies using the CAL-27 OSCC cell line, quercetin inhibited glycolysis and proliferation of the cells by inactivating the G3BP1/YWHAZ axis [92] and suppressed cell invasion by activating the miR-1254/CD36 signaling pathway [93]. Another in vitro study demonstrated that quercetin selectively induced cell cycle arrest in OSCC cell lines but had no effect on human keratinocytes. Moreover, quercetin suppressed cancer cell metastasis via an EMT-mediated pathway [94]. Although promising laboratory results have demonstrated the therapeutic potential of quercetin, there are no clinical trials to date; hence, its relevance to patient outcomes remains speculative.

Isothiocyanates (ITCs) are produced by enzymatic conversion of metabolites called glucosinolates. Glucosinolates are rich in cruciferous vegetables, such as broccoli, cabbage, cauliflower, and kale. Phenethyl isothiocyanate (PEITC), allyl isothiocyanate (AITC), benzyl isothiocyanate (BITC), and sulforaphane (SFN) have been studied for their chemopreventive role [72,95]. A blinded, randomized, placebo-controlled trial analyzed the safety and efficacy of PEITC, in the form of nutri-PEITC jelly, in oral or oropharyngeal cancer. The group administered 20 mg of PEITC (in 200 g of nutrient jelly) for 3 months showed improved health-related quality of life, stable disease, and longer progression-free survival than the control group (*p* < 0.001). Severe adverse events were not noticed [96]. Several in vivo studies have revealed underlying molecular mechanisms. In a mouse xenograft model of oral cancer, PEITC increased the levels of the oxidative DNA damage markers 8-oxo-dG and p53. The researchers also revealed that PEITC induced ROS formation and cell cycle arrest in the G1/S phase in vitro, suggesting that PEITC triggers ROS-mediated cell cycle arrest [97]. Another in vivo study found that PEITC induced apoptosis by upregulating tumor necrosis factor-related apoptosis-inducing ligand (TRAIL), death receptor (DR) 4, and DR5 [98]. Multiple studies have highlighted the potential of AITC and BITC in HNSCC treatment through the regulation of apoptosis, cell cycle arrest, cell migration, and chemotherapy sensitivity [99]. For example, AITC reduced cell viability by activating transient receptor potential ankyrin1 (TRPA1) receptor in human OSCC [100]. Moreover, in human CAL-27 cisplatin-resistant oral cancer cells, AITC suppressed the Akt/mTOR proliferative signaling pathway and induced apoptosis via caspase-9 and caspase-3 [101]. An in vitro study of multiple HNSCC cell lines indicated that BITC inhibited the expression of the EMT marker vimentin, decreased cell migration, and increased cisplatin toxicity [102]. Lan et al. demonstrated that SFN activated the NRF2 pathway, thereby inhibiting oxidative stress-associated carcinogenesis in oral cancer cells [103]. In an HNSCC human cell line, treatment with broccoli extract, which is rich in SFN, increased the cytotoxicity of cisplatin two-fold, and that of 5-fluorouracil ten-fold, while the combinations had little effect on noncancerous cells [104]. In a pilot study of 10 healthy subjects, SFN-rich broccoli sprout extracts showed high bioavailability [105], supporting the potential of SFN as a safe and efficient therapeutic compound for HNSCC.

Resveratrol is a phytoalexin with antioxidant, antimicrobial, and antiinflammatory activities and is abundant in grapes, berries, and peanuts [106]. Its chemopreventive and therapeutic potential in HNSCC is mediated by diverse molecular pathways, as demonstrated by several preclinical studies. A recent meta-analysis of five in vivo studies of oral cancer concluded that resveratrol has the potential to suppress cancer cell proliferation and drive neoplastic apoptosis. A statistically significant reduction was demonstrated in neoplastic parameters, with an overall effect size of 0.85 (95% Cl 0.74–0.98) [107]. Fukuda et al. showed that resveratrol induced selective autophagy in human OSCCs by blocking sterol regulatory element binding protein 1 (*SREBP1*) gene expression and suppressing lipid metabolism [108]. One preclinical study found that resveratrol targeted tumor-initiating stem-like and EMT properties. The expression of stemness gene signatures (Oct4, Nanog, and Nestin) and EMT markers (Slug, ZEB1, N-cadherin, and vimentin) decreased [109]. Furthermore, Tyagi et al. demonstrated that resveratrol selectively induced DNA damage, cell cycle inhibition, and apoptosis, independent of Smad4 status. Loss or alterations in Smad4 signaling leads to genomic instability in HNSCC, broadening its applicability in diverse HNSCC cases [110]. Resveratrol has also demonstrated the ability to enhance the efficacy of standard cancer therapies. Resveratrol enhanced cisplatin sensitivity in HNSCC cell lines, which was linked to increased *MYC* and *TP53* expression and decreased *BCL-2* expression. It also reduced cisplatin toxicity in normal adherent cells [111]. Similarly, another study reported enhanced cisplatin and radiation sensitivity in HNSCC xenograft nude mouse models through upregulation of regenerating gene III (*REGIII*) expression, further emphasizing its potential as an adjunct therapy [112]. Resveratrol has also been studied in combination with other dietary antioxidants. When administered with curcumin, resveratrol had a synergistic antitumor effect, compared to when each was administered separately. The combination increased PARP cleavage, Bax/Bcl-2 ratio, cytoplasmic NF-κB accumulation, and autophagy vacuoles. This combination treatment inhibited ERK1 and ERK2 phosphorylation [113]. Moreover, combination treatment with EGCG synergistically induced apoptosis in HNSCC cell lines via caspase-3 and PARP cleavage in vitro. In addition, the inhibition of the Akt/mTOR signaling pathway was observed both in vitro and in vivo in HNSCC-xenografted nude mice. Xenografted tumor volume and weight were significantly reduced [114]. Further clinical studies are necessary to validate the efficacy of resveratrol and to establish optimal dosing strategies for its integration into standard HNSCC care.

Luteolin is a flavonoid that has been extensively studied in various types of cancer treatments. Several studies have found the antitumor effect of luteolin relates to integrin β1. Integrin β1 is a key molecule involved in cancer progression, angiogenesis, invasion, and therapeutic resistance. Integrin β1 enhances VEGF signaling, which is essential for angiogenesis [115], and interacts with CD147 [116] and PLOD2 [117] in laryngeal cancer proliferation. Integrin β1 was also revealed to regulate perineural invasion and radio resistance of OSCC [118]. A recent preclinical study has demonstrated that luteolin enhanced tumor suppression and angiogenesis of laryngeal cancer cells during radiotherapy by downregulating integrin β1 [115]. Luteolin-enhanced radiation sensitivity has also been observed in an in vitro study of oral cancer stem cells. In this study, luteolin was found to inhibit IL-6/STAT3 signaling, which is associated with cancer stem-like properties and progression [119]. An in vivo study using an HNSCC xenograft mouse model showed that luteolin treatment reduced tumor growth. Luteolin treatment disrupted gene expression and microRNA profiles, thereby promoting tumor suppression [120]. Another in vivo study used a water-soluble polymer-encapsulated nano-luteolin to overcome low bioavailability and systemic delivery. Nano-luteolin had a lower half maximal inhibitory concentration than luteolin and had a greater inhibitory effect on tumor growth [121]. These findings demonstrate the promise of luteolin as a protective anticancer agent. However, most findings are based on preclinical models; clinical evidence is scarce and novel approaches to improve its bioavailability in clinical settings are important. Therefore, further clinical studies are warranted.

**Table 1:** Possible preventive or therapeutic dietary antioxidants in HNSCC.

Dietary Antioxidants	Diet Source	Study Design	Mechanism or Effect	Authors (Years)
Vitamin C (ascorbate)	Citrus fruits, broccoli, bell peppers, strawberries	Prospective cohort study (120,852 subjects)	Lower the risk of HNSCC subtypes	Munter et al. (2015) [48]
		Pooled analysis of 12 case–control studies (7002 cases)	Lower the risk of HNSCC subtypes	Li et al. (2012) [45]
		Pooled analysis of 10 case–control studies (5959 cases)	Lower the risk of HNSCC subtypes	Edefonti et al. (2015) [46]
		Case–control study (101 patients)	Lower the risk of HNSCC subtypes	Saka-Herrán et al. (2023) [47]
Vitamin E (tocopherols and tocotrienols)	Peanuts, sunflower seeds, almonds, olive oil, spinach	Pooled analysis of 10 case–control studies (5959 cases)	Lower the risk of HNSCC subtypes	Edefonti et al. (2015) [51]
		Randomized controlled trial (60 patients)	Reduce the severity and duration of radiation-induced oral mucositis and dysphagia when given with pentoxifylline	Sayed et al. (2019) [56]
		Double-blinded randomized placebo-controlled trial (45 patients)	Protective effect against radiotherapy-induced xerostomia when given with vitamin C	Chung et al. (2016) [57]
		Double-blinded randomized controlled trial (54 patients)	Decrease the incidence of radiation-induced oral mucositis	Ferreira et al. (2004) [58]
Carotenoids	Fruits and vegetables, mainly yellow to red (pumpkin, carrot, spinach, tomato, tangerine)	Pooled analysis of 10 case–control studies (5959 cases)	Lower the risk of HNSCC subtypes	Leoncini et al. (2016) [65]
		Meta-analysis of 15 case–control studies and one prospective cohort study	Lower the risk of HNSCC subtypes	Leoncini et al. (2015) [66]
		Pooled analysis of five case–control studies (2452 cases)	Lower the risk of HNSCC subtypes	Edefonti et al. (2012) [67]
		Randomized controlled trial (540 patients)	Decrease local recurrence and severe adverse effects of radiation therapy	Meyer et al. (2007) [55]
Epigallocatechin-3-gallate	Green tea	Case–control study (147 patients)	Reduce the risk of HNSCC	Rafieian et al. (2019) [73]
		Case–control study (396 patients)	Reduce the risk of HNSCC	Huang et al. (2014) [74]
		Phase Ib clinical trial (19 patients)	Response in advanced premalignant lesions when given with erlotinib	Shin et al. (2020) [77]
		Phase II randomized, placebo-controlled trial (39 patients)	Response in oral premalignant lesions	Tsao et al. (2009) [78]
		Single-blind, randomized, controlled trial (61 patients)	Mouthwash improves oral health in patients with oral cancer	Liao et al. (2021) [79]
		In vivo study of HNSCC stem cell xenograft mouse model	Suppress stem cell markers by inhibiting Notch pathway and augment cisplatin sensitivity	Lee et al. (2013) [80]
Curcumin	Turmeric	Meta-analysis of six randomized controlled trials (266 patients)	Prevent and ameliorate therapy-induced oral mucositis and weight loss	Zhang et al. (2021) [88]
		Randomized double blind placebo-controlled phase IIb (223 patients)	Clinical and histological response in oral leukoplakia	Kuriakose et al. (2016) [87]
		Randomized double-blind placebo-controlled phase I trial (12 patients)	Decrease inflammatory markers and *Bacteroides* species in saliva and increase immune T cells	Basak et al. (2020) [85]
		Pilot clinical trial (15 patients)	Decrease factors involved in angiogenesis and cell invasion, such as FGF-2, GM-CSF, and IL-7	Latimer et al. (2015) [84]
		Pilot clinical trial (39 patients)	Suppress tumor progression by reducing IKKβ activity	Kim et al. (2011) [122]
		Systematic review of 30 in vitro and in vivo studies of HNSCC cell lines	Induce cytotoxicity, apoptosis, cell cycle arrest	Borges et al. (2017) [82]
Quercetin	Onion, kale, caper	In vitro study of OSCC line	Induces ferroptosis by inactivating mTOR/S6KP70 pathway	Zhu et al. (2024) [89]
		In vitro study of OSCC line	Induce cell cycle arrest and apoptosis by activating p38 pathway	Son et al. (2023) [90]
		In vitro study of tongue SCC cell line	Induce apoptosis via the JNK activation-regulated ERK/GSK-3α/β-mediated mitochondria-dependent apoptotic signaling pathway	Huang et al. (2022) [91]
		In vitro study of OSCC line	Inhibit glycolysis and cell proliferation by inhibiting G3BP1/YWHAZ axis	Hu et al. (2023) [92]
		In vitro study of OSCC line	Inhibit cell survival and invasion via miR-1254/CD36 cascade	Chen et al. (2021) [93]
		In vitro study of OSCC line	Inhibit cell survival and metastasis by inhibiting TGF-β1 inducing EMT	Kim et al. (2020) [94]
Isothiocyanates	Cruciferous vegetables	Randomized blinded placebo-controlled trial (72 patients)	Stabilize disease, improve QoL and PFS in patients with oral and oropharyngeal cancer	Lam-Ubol et al. (2023) [96]
		In vitro study of OSCC line and in vivo study of OSCC xenograft mice model	Induce ROS-mediated cell cycle arrest	Lam-Ubol et al. (2018) [97]
		In vivo study of OSCC xenograft mice model	Induced apoptosis by enhancing TRAIL and upregulating DR4 and DR5	Yeh et al. (2015) [98]
		In vitro study of OSCC line	Reduce cell viability by activating TRPA1 receptor	Kiss et al. (2022) [100]
		In vitro study of OSCC line	Induce apoptosis by inhibiting Akt/mTOR pathway and enhancing caspase-3 and caspase-9 in cisplatin-resistant OSCC	Chang et al. (2021) [101]
		In vitro study of HNSCC cell line	Inhibit cell migration and increase cisplatin sensitivity	Wolf et al. (2014) [102]
		In vitro study of human keratinocytes and in vivo study of mouse tongue	Inhibit oxidative stress-associated oral carcinogenesis by activating NRF2 pathway	Lan et al. (2016) [103]
		In vitro study of HNSCC cell line	Increase cisplatin and 5-FU cytotoxicity	Elkashty et al. (2018) [104]
Resveratrol	Grapes, red wine, berries, peanuts	Systematic review and meta-analysis of five in vivo studies of oral cancer cells	Suppress tumor growth and induce apoptosis by activating various pathways	Alam et al. (2024) [107]
		In vitro study of OSCC cell line and in vivo study of xenograft mouse model	Induce autophagy by blocking *SREBP1* expression	Fukuda et al. (2022) [108]
		In vitro study of HNSCC line	Increase cisplatin sensitivity by inducing apoptosis and *TP53*, *BCL-2*, and *MYC* modulation	Bostan et al. (2021) [111]
		In vivo study of HNSCC xenograft mouse model	Increase cisplatin and radiation sensitivity by enhancing REGIII expression	Mikami et al. (2019) [112]
		In vitro study of HNSCC cell line and in vivo study of xenograft mouse model	Reduce tumor initiating stem-like and EMT properties	Hu et al. (2012) [109]
		In vitro study of HNSCC cell line and in vivo study of xenograft mouse model	Induce selective DNA damage, cell cycle arrest, and apoptosis independent of Smad4 status	Tyagi et al. (2011) [110]
Luteolin	Chamomile tea, celery, parsley	In vitro study of laryngeal cancer cells and in vivo study of xenograft mouse model	Enhance radiation sensitivity by downregulating integrin β1	Li et al. (2023) [115]
		In vitro study of oral cancer stem cells	Enhance radiation sensitivity and inactivate IL-6/STAT3 signaling	Tu et al. (2016) [119]
		In vivo study of HNSCC xenograft mouse model	Reduce tumor growth by inhibiting histone acetylation	Selvi et al. (2015) [120]
		In vitro and in vivo study of HNSCC cell line	Inhibit tumor growth	Majumdar et al. (2014) [121]

Abbreviations: HNSCC, head and neck squamous cell carcinoma; FGF-2, fibroblast growth factor-2; GM-CSF, granulocyte-macrophage colony stimulating factor; IL, interleukin; IKKβ, IκB kinase β; OSCC, oral squamous cell carcinoma; mTOR, mammalian target of rapamycin; JNK, c-Jun N-terminal kinase pathway; ERK, extracellular signal-regulated kinase; GSK, glycogen synthase kinase; G3BP1, ras-GTPase-activating protein SH3 domain-binding protein 1; YWHAZ, tyrosine 3-monooxygenase/tryptophan 5-monooxygenase activation protein zeta (14-3-3ζ); TGF-β1, transforming growth factor-β1; QoL, quality of life; PFS, progression-free survival; TRAIL, tumor necrosis factor-related apoptosis-inducing ligand; DR, death receptor; TRPA1, transient receptor potential ankyrin 1; Akt, protein kinase B; NRF2, nuclear factor erythroid 2-related factor 2; 5-FU, 5-fluorouracil; *SREBP1*, sterol regulatory element-binding protein1; *TP53*, tumor protein p53; *BCL-2*, B-cell leukemia/lymphoma 2 protein; *REGIII*, regenerating islet-derived III; EMT, epithelial-mesenchymal transition; DNA, deoxyribonucleic acid; Stat-3, signal transducer and activator of transcription-3.

## 4. Conclusions

Dietary antioxidants have been proposed as potential modulators of oxidative stress and may offer chemopreventive or therapeutic benefits by neutralizing reactive oxygen species (ROS), influencing redox-sensitive signaling pathways, and modulating the tumor microenvironment.

In this review, we discussed nine dietary antioxidants—some of which, including vitamins C and E, carotenoids, epigallocatechin-3-gallate, and curcumin, have shown possible benefits in preclinical and early clinical studies. Others, such as quercetin, isothiocyanates, resveratrol, and luteolin, remain primarily supported by experimental evidence. However, despite this growing body of research, the current clinical application of dietary antioxidants in HNSCC remains limited. Notably, many studies in this field rely on observational designs, small sample sizes, or self-reported dietary data, often lacking standardized protocols or long-term follow-up. While some large cohort studies and meta-analyses have suggested potential associations between antioxidant intake and reduced cancer risk, these findings are often undermined by methodological limitations and inconsistencies. As such, the relationship between dietary antioxidants and clinical outcomes in HNSCC should be regarded as plausible but not yet definitive. Moreover, challenges, such as inconsistent clinical trial outcomes, variations in bioavailability, and the need for tailored dosing regimens, must be addressed to fully realize their therapeutic potential.

It is also important to acknowledge that the role of antioxidants in cancer remains complex and, at times, contradictory, as certain antioxidant mechanisms may exhibit both tumor-suppressive and tumor-promoting effects depending on the context. Given these uncertainties, our review does not intend to assert conclusive therapeutic recommendations but rather to provide a mechanistic and evidence-based overview of dietary antioxidants that have demonstrated potential in modulating oxidative stress. Future research should prioritize well-designed randomized clinical trials, mechanistic studies in human models, and strategies to enhance the bioavailability and delivery of promising compounds. By integrating these efforts into a multidisciplinary approach, dietary antioxidants could potentially offer safe, accessible, and effective strategies for the prevention and management of HNSCC.
